# Notes and News

**Published:** 1893-02-11

**Authors:** 


### NOTES AND NEWS.

COLLECTED AND COMMUNICATED.

THE much discussed proposal to erect an infirmary in Tollington Park for the inhabitants of Islington has been finally rejected by the Board of Guardians by a majority of one, after having gained the assent of the Local Government Board. The infirmary was to have accommodated 750 patients, the cost of erection being estimated at £100,000. The rejection of the scheme gives satisfaction, at any rate, to the dwellers in the vicinity of Tollington Park.

THE friends of the G.F.S. remember its interest in a very practical manner. Another convalescent home is to be started for their benefit, through the generosity of Mrs. Hervey, a lady residing in the Isle of Wight. She has offered the Diocesan Council of the Society a commodious building, situated in that charming spot, Shanklin, in the Isle of Wight. A meeting was held to consider the possibility of carrying out the project entailed by Mrs. Hervey's offer. The general feeling was that no difficulty would be encountered in providing the requisite means, and the gift was therefore gratefully accepted on behalf of the Society.

THE last quarterly court of the Radcliffe Infirmary revealed a most unfortunate financial condition. The adverse balance at the end of the year amounts to £1,400, and subscriptions have fallen off. We prophesy a better state of things with a better building. The result of an adequate provision tor the sick is very encouraging, as experienced by the Liverpool Infirmary, where subscriptions have increased by £300, and we doubt not that a good infirmary at Oxford will receive corresponding support. The authorities have decided on expending £600 in providing another small addition to the institution.

A NEW hospital, l'Hôpital International, has just been opened in the Rue de la Santé, Paris. This hospital, which is free, is intended to meet the wants of patients from the provinces and foreigners besides those of the inhabitants of the city. The utmost care has been taken in the sanitary arrangements of the institution. The operating theatre is especially complete. Ample light is afforded, and the electric light laid on ready to the surgeon's hand. All water for use is sterilised and filtered, and the hot water is regulated to the required temperature. All the departments and wards have been constructed with rounded corners. The heating and ventilation are reported not to be quite as perfect as might have been desired. Dr. Pean has attached a policlinic to the hospital.

WE are glad to hear that the authorities of the Royal Infirmary, Newcastle, have determined to publish their accounts in the form adopted by the Metropolitan Hospital Sunday Fund, after conference with a committee of secretaries. We congratulate Mr. Redmayne, the house governor, on the public spirit he has shown in this matter, and hope that the example of the Newcastle Infirmary will be followed throughout the country.

WE are glad to learn that in all probability better things will obtain before very long at the Royal Hospital for Incurables, Putney Heath. The first meeting of a committee, under the presidency of Lord Aberdeen, has already been held to consider the recommendations contained in the report of the Lords' Committee on Metropolitan Hospitals concerning this institution. The time has passed when large endowments or large revenue from any sources is to exempt institutions from conforming to modern requirements in regard to management and other matters.

THE anti-vaccination movement has received a severe blow from the action of the inhabitants of Warrington, where small-pox has lately been rife. The belief in the immunity afforded by vaccination suddenly seized the town, and on one occasion a crowd of four or five hundred persons is stated to have been assembled at one time at the vaccination station demanding immediate attention. More convincing proof than this panic action of the efficacy of vaccination as affording either immunity from small-pox, or the minimising of its severity, may be found in the exhaustive report of the German Royal Commission on small-pox mortality for 1890.

MR. LAWRENCE H. WATSON, Secretary to the Glasgow Sick Poor and Private Nursing Association, was killed last week whilst out hunting, by the falling of his horse at a "double." Mr. Watson was a practised rider, but rode a young animal untrained to fencing. The Association of which he was so many years the indefatigable Secretary has sustained a painful loss, and the sick poor of Glasgow have to mourn the death of a sincere and tried friend. Mr. Watson, who was a member of the firm of Morres, Carson, and Watson, was a highly respected citizen of Glasgow, and his untimely death at the early age of forty has evoked universal sympathy.

WE heartily endorse the many words uttered in praise of the Liverpool Royal Infirmary by the speakers at its annual meeting last week, even if we do not go so far as did the American authority who said that "The Liverpool Infirmary is to-day probably the best hospital in the world." This is very high and generous praise from one who must be well acquainted with the John Hopkins Hospital. The record of the past year is a good one. The building account of the new infirmary is paid off; the number of patients has risen from 2,926 to 3,143, and annual subscriptions have increased by £300. Still the income of the institution remains inadequate to cover the expenditure, and Mr. Ismay, president on the occasion, in an admirable speech showed the necessity for further assistance from the public, and the importance of establishing an endowment fund. He further illustrated the desirability he attached to such a step by presenting the magnificent sum of £1,000 towards it. The people of Liverpool have built their magnificent hospital at a cost of upwards of £170,000, and therefore we do not doubt that they will provide maintenance in an equally generous spirit.

